# Supplementary material for: No evidence for adult smartphone use affecting attribution of communicative intention in toddlers: Online imitation study using the Sock Ball Task
Source: PLoS One. 2024 Mar 22;19(3):e0300874. doi: 10.1371/journal.pone.0300874 (PMC10959379; doi:10.1371/journal.pone.0300874)
Supplement: S1 Appendix — (DOCX) [file pone.0300874.s001.docx]

**S1 Appendix. The faithful imitation score preregistered for Experiment 1.**

# Coding

The preregistered faithful imitation score ranged from 0 to 8 and was a sum of the action performance score and the order score, each ranging from 0-4. The action performance score reflected the number of types of action steps (cover, grasp, move, drop) that the toddler produced. We also coded the order in which each type of action step (and the goal-outcome: ball-on-paper) was produced for the first time. For each child, this resulted in a series of codes that had a length between 0 (none of the target behaviors produced) and 5 (e.g., cover, grasp, move, drop, ball-on-paper; or ball-on-paper, grasp, cover, move, drop). The order score was calculated by assigning one point for each consecutive pair in the series of codes corresponding to the order of the actions in the demonstration stimuli. A score of 1 was given even if the actions in the pair were not consecutive actions in the demonstration video. Thus, e.g., a pair cover-move earned 1 point even though in the demonstration video, the sequence was cover-grasp-move. In defining the order score, we followed the scoring rules of some previous studies [1, 2].

# Results

For the statistical analysis, we used SPSS (Statistical Package for Social Sciences 28.0). The preregistered faithful imitation score deviated significantly from the normal distribution, as indicated by a Kolmogorov-Smirnov test, *D*(48) = 0.268, *p* < .001. Moreover, the skewness value was 1.28, indicating a right-skewed distribution. Therefore, a non-parametric test was conducted in the analysis, and a *p*-value for two-tailed test was reported.

There was no statistical difference between the smartphone (*Mdn* =.50, range = 0-5) and the wristwatch condition (*Mdn* = 1, range = 0-7), Mann Whitney *U*(*N*_smartphone._ = 24, *N*_wristwatch._ = 24) = 234.50, *z* = -1.16, *p* = .252, *r* = -.17.

# Interpretation

The analysis found no evidence that toddlers who watched the demonstration disrupted by smartphone use imitated the modeled means less faithfully than those who watched the demonstration disrupted by fiddling with a wristwatch. Thus, the analysis based on the preregistered definition of the faithful imitation score led to the same conclusion as the analysis using the score reported in the main manuscript for Experiment 1 and later preregistered for Experiment 2.

# Discussion

Our reasons for abandoning the preregistered definition were the following. After having coded the toddlers’ behaviors in Experiment 1 we realized that the preregistered score was bound to systematically underestimate the faithful imitation of those toddlers, who improved fidelity of their imitation during the testing session. This is because the preregistered score relied specifically on the order in which each target behavior was produced *for the first time*. Consequently, it would fail to generate higher scores for the babies, who first produced some elements of the modeled sequence and later produced them in the correct (or at least improved) order, than for children who produced the same target behaviors but never improved in producing them in the correct order. For instance, a toddler might have started by grasping the sock ball and placing it on the paper but later grasped the ball again through a paper and placed it on the paper again. This improvement in reproducing faithfully the order would not be captured by the score based on the preregistered definition. To amend this, we changed the definition of the faithful imitation score to one based on the best sequence of the target elements produced by the child during the test phase. For details, see the Methods section of Experiment 1 in the main manuscript.

# References

1. Bauer PJ, Hertsgaard LA, Dropik P, Daly BP. When even arbitrary order becomes important: Developments in reliable temporal sequencing of arbitrarily ordered events. Memory. 1998;6(2):165-98.

2. de Haan M, Bauer PJ, Georgieff MK, Nelson CA. Explicit memory in low-risk infants aged 19 months born between 27 and 42 weeks of gestation. Developmental Medicine & Child Neurology. 2000;42(5):304-12.
