# Supplementary material for: No evidence for adult smartphone use affecting attribution of communicative intention in toddlers: Online imitation study using the Sock Ball Task
Source: PLoS One. 2024 Mar 22;19(3):e0300874. doi: 10.1371/journal.pone.0300874 (PMC10959379; doi:10.1371/journal.pone.0300874)
Supplement: Attachment — Submitted filename: Response to Reviewers.docx [file pone.0300874.s002.docx]

|  | **Faculty of Health Sciences / Department of Psychology / Research Group on Child Development**  Your reference: PONE-D-23-31320R2  Date: 04.03.2024 |
| --- | --- |
| Dr Giulia Ballarotto  Academic Editor  PLOS ONE | |
|  | |

Response to Reviewer

Dear Dr Ballarotto,

Thank you for your letter and for inviting us to resubmit our manuscript after minor revision. Please see below a quick rundown of how we addressed the reviewer’s feedback:

*The study addresses a central issue in this historical period, allowing for reflection on significant social changes by considering dyadic interaction. I find both studies to be well-structured, and the paper is well-written.*

We thank the Reviewer for this positive feedback.

*However, I believe that the introduction and discussions should further address the competencies and needs of children in this specific age group, as well as the parental role (particularly scaffolding) that could help further support the emerged results. I recommend considering some interesting studies on the topic:*

*Ballarotto, G., Murray, L., Bozicevic, L., Marzilli, E., Cerniglia, L., Cimino, S., & Tambelli, R. (2023). Parental sensitivity to toddler’s need for autonomy: An empirical study on mother-toddler and father-toddler interactions during feeding and play. Infant Behavior and Development, 73, 101892.*

*Andreadakis, E., Laurin, J. C., Joussemet, M., & Mageau, G. A. (2020). Toddler temperament, parent stress, and autonomy support. Journal of Child and Family Studies, 29, 3029-3043.*

*Zhang, H., & Whitebread, D. (2021). Identifying characteristics of parental autonomy support and control in parent–child interactions. Early Child Development and Care, 191(2), 307-320.*

*Ballarotto, G., Cerniglia, L., Bozicevic, L., Cimino, S., & Tambelli, R. (2021). Mother-child interactions during feeding: A study on maternal sensitivity in dyads with underweight and normal weight toddlers. Appetite, 166, 105438.*

*Lincoln, C. R., Russell, B. S., Donohue, E. B., & Racine, L. E. (2017). Mother-child interactions and preschoolers’ emotion regulation outcomes: Nurturing autonomous emotion regulation. Journal of Child and Family Studies, 26, 559-573.*

As per Reviewer’s suggestion we addressed the link with the topics and papers raised by the Reviewer *both* in the Introduction and in the Discussion.

In the Introduction we added a whole short separate paragraph that points to the importance of the topics raised by the Reviewer in connection with our study, while acknowledging that giving them full justice lies beyond the scope of the current paper (lines: 188-193 in the clean manuscipt).

Potential impact of parental smartphone use on toddler emotional development and the interplay with factors such as parental stress and support of the child’s autonomy on one hand, and children’s needs, temperament and emotional competencies on the other remain an important topic for future research. However, it lies beyond the scope of the current paper. This broad topic and specific literature (84-88) were brought to our attention by an anonymous reviewer. We come back to it briefly in the discussion.

In the discussion, we added new text where we acknowledge that prior experience with parent scaffolding might have contributed to the lack of predicted effect in toddlers in our study. We thank the Reviewer for pointing us towards this possibility (lines: 765-769 in the clean manuscript)..

[…] Relatedly, children’s past experiences with how parents respond to their behavior in emotionally demanding situations, such as parental scaffolding and autonomy support, might foster emotional self-regulation (e.g., ability to wait) when children encounter similar events in the future (85, 88, 124).

We used this opportunity to proofread the manuscript again and corrected minor typos and errors. We hope that with this minor revision the manuscript now finds your acceptance for publication in PLOS ONE.

Sincerely,

| Mikolaj Hernik  Associate Professor  mikolaj.l.hernik@uit.no  +47 77 62 52 59 |  |
| --- | --- |
